# The Chinese version of the family accommodation scale for obsessive-compulsive disorder self-rated: reliability, validity, factor structure, and mediating effect

**DOI:** 10.3389/fpsyt.2022.970747

**Published:** 2022-08-11

**Authors:** Zhenhua Liao, Lijun Ding, Ciping You, Ying Chen, Wenchang Zhang

**Affiliations:** ^1^School of Public Health, Fujian Medical University, Fuzhou, China; ^2^Xiamen Xianyue Hospital, Xiamen, China; ^3^School of Health, Fujian Medical University, Fuzhou, China

**Keywords:** family accommodation, self-rated, reliability, validity, mediating effect

## Abstract

**Background:**

Family accommodation (FA) in obsessive compulsive disorder (OCD) is a common phenomenon. Based on the cost of training interviewers and the time required to administer the scale, the Family Accommodation Scale for Obsessive-Compulsive Disorder Interviewer-Rated (FAS-IR) has been restricted to specific settings. A self-rated version of the family accommodation scale may solve these problems. The aim of this study was to examine the reliability, validity and factor structure of the Family Accommodation Scale Self-rated version (FAS-SR), and the relationship among FA, symptom severity and functional impairment.

**Methods:**

In total, 171 patients with OCD and 145 paired relatives participated in this study. The Sheehan Disability Scale (SDS), Obsessive-Compulsive Inventory Revised (OCI-R), Zung Self-Rating Depression Scale (Zung-SDS), 12-item Family Assessment Devices (FAD-12), Clinical Global Impression of Severity Scale (CGI-S), Global Assessment of Functioning (GAF), and Yale-Brown Obsessive-Compulsive Scale (Y-BOCS) were used as tools for patients. The FAS-SR, FAS-IR, FAD-12, and the patients’ symptom severity of Y-BOCS compulsion were used as tools for relatives. The psychometric properties of the FAS-SR were evaluated using Cronbach’s alpha coefficient, test-retest reliability and validity. Mediation analysis was used to determine the relationship among FA, symptom severity and functional impairment.

**Results:**

A total of 97.9% of relatives of OCD patients reported at least one kind of FA behavior, and 56.6% of participants engaged in FA every day in the past week. The FAS-SR includes a three-factor structure: (1) providing reassurance and participation; (2) facilitation; and (3) modification. The scale’s Cronbach’s alpha and test-retest coefficients were 0.875 and 0.970, respectively. The total FAS-SR score was significantly positively associated with the Y-BOCS, FAD-12, CGI-S, FAS-IR, and SDS scores, and negatively associated with the total GAF score. FA partially mediated the relationship between symptom severity and functional impairment.

**Conclusion:**

The FAS-SR was proven to have satisfactory psychometric properties, and can play an important role in the evaluation and early intervention of OCD. This result indicates the importance of assessing symptom severity in conjunction with FA when evaluating OCD patients’ functional impairment.

## Introduction

Obsessive-compulsive disorder (OCD) is relatively prevalent among mental disorders and has a lifetime prevalence of 2.4% in China, according to a recent national epidemiological study (1). In addition, OCD was estimated to strongly contribute to the global burden of disease (2). OCD is a chronic, prolonged, serious and disabling disorder that frequently interferes with individuals’ ability to function in society and decreases their quality of life (3–6). The negative or adverse consequences of OCD are not limited to patients alone (7, 8). Their family members, including parents, spouses, siblings and significant others, are also affected and distressed by symptoms in both adult and pediatric OCD patients, which cause unpleasant experiences and create a great burden for their caregivers. Patients’ symptoms and behaviors play an important role in the course of the disorder and treatment outcomes (9, 10). According to recent research, it is clear that in addition to the symptoms of OCD that affect patients, their family members’ responses have a deleterious effect on treatment outcomes (9–14).

In the last two decades, the relationship between OCD disorders and family dynamics has attracted increasing attention from researchers, and awareness of family accommodation (FA) has aroused growing interest in the illustration of OCD etiology and treatment outcomes (15–17). The terminology of FA refers to family members participating and assisting in the patients’ rituals and accommodating their compulsions to prevent and alleviate their anxiety, which are behaviors frequently observed and reported in the families of both adult and pediatric OCD patients (8). On the basis of previous reports, almost all family members of OCD patients frequently experience this phenomenon on a daily basis or in extreme situations (7, 8). Accommodating behaviors can be maladaptive responses to OCD, even if FA is often treated as a global construct. The primary forms of FA included providing verbal reassurance, refraining from saying or doing things to trigger behaviors, participating in and facilitating compulsions, and following and respecting the rigid rules established by patients. The original intention of family members of OCD patients was an attempt to relieve their loved ones’ anxiety and distress, and perhaps accelerate the compulsive behavior process, while their responses might be “successful” in the short term, the behaviors are maintained and repeated later (10, 18). As a result, FA behaviors actually prevent patients from confronting their obsessions, compulsions and anxiety. Furthermore, the patient’s symptoms ultimately expand seriously, and an escalating loop between OCD symptoms and FA behaviors is established.

Selective serotonin reuptake inhibitors (SSRIs) and exposure-based cognitive-behavioral therapy (CBT) make up the standard first-line pharmacotherapy and psychotherapy options for OCD treatment (6, 19, 20). However, approximately half of individuals with OCD do not benefit from standard treatments and become refractory (21, 22). Factors associated with poor response to treatment of OCD have been widely reported, and there is some consensus among healthcare providers regarding these factors. For example, FA has been associated with poor treatment response in both adult and pediatric OCD patients, hindering the goals of CBT treatment and serving as an obstacle to the improvement of symptoms and family functioning in both pharmacological and psychotherapy regimens (10, 11, 13). Thus, the reduction of FA is increasingly referred to as an important part of the treatment plan and clinical target for OCD patients and even serves as a possible mediating factor of treatment outcomes (9, 12, 23, 24). As a result, the integration and management of FA as a plan to treat OCD patients could further advance the knowledge of OCD and improve clinical outcomes. In addition, research on FA will contribute to clinicians’ understanding of the recognition, assessment and treatment outcomes of OCD.

Based on the abovementioned definition and various manifestations, several instruments have been developed to measure and individually assess FA by the pattern method of evaluation based on relatives’ reports on the Family Accommodation Scale Interviewer-Rated (FAS-IR) and Family Accommodation Scale for OCD Self-Rated version (FAS-SR) (18, 25–27). The FAS-IR was originally developed by Calvocoressi et al. and was improved, revised and readjusted from the 13-item FAS reported in 1999 (18). The FAS-IR was regarded as the gold standard inventory to measure FA behaviors, and has been adapted and translated into Brazilian Portuguese and Chinese versions (26, 28). The scale is extensively used in clinical and research settings and has demonstrated strong psychometric features (18, 26, 28). Unfortunately, some common disadvantages limit the use of clinician-administration instruments. First, it is costly and time-consuming to apply the instruments due to interviewer training and instrument administration. Second, it may miss some important information if the interviewees are unwilling to admit and report their responses to the OCD family member in the interviewer-rater investigation, especially when they realize that the patients’ behaviors and/or requests were unreasonable. Third, the interviewers may easily recognize the distributed group in face-to-face interviews even if a blinded method is used in the random control study. As a result, the self-report questionnaire for assessing FA will improve the corresponding items and evaluate the occurrence or incidence of FA in a targeted manner by retaining the overall structure of the FAS-IR and refining the items. Compared with the clinician-rated instrument, the FAS-SR addresses these shortcomings and facilitates a more widespread collection of FA data. Additionally, the FAS-SR refers to family members who can independently measure and evaluate the incidence of FA according to the standard items and some examples, and the evaluation result is usually easy to understand.

Although FA in the OCD population has a relatively high incidence globally, individualized assessment of FA and associated factors related to treatment response is required in China (8). Similar to Western countries and other Asian countries, FA frequently occurs in family members of OCD patients in China according to our previous report and clinical experience (28, 29). However, few studies have emphasized OCD-related family pathology in the Chinese population. Although our previous study reported the Chinese version of the FAS-IR (28), the lack of these FAS-SR studies led to a lag in the development of family therapy and intervention for OCD patients, especially in regions with a paucity of trained clinical professionals. Therefore, the development and adaptation of the Chinese version of the FAS-SR will allow clinicians to observe and quantify the frequency and types of FAs in Chinese OCD patients and easily observe their associations with illness and as treatment-related variables based on relatives’ understanding and realization.

This primary aim of this study is based on the abovementioned research in three ways. First, this study assessed the incidence of FA and examined the reliability and validity of the Chinese version of the FAS-SR. We hypothesized that the frequent incidence of FA behavior is based on the evaluation of the FAS-SR in individuals with OCD in China. Moreover, we hypothesized that the total score of the FAS-SR would be strongly correlated with the FAS-IR, which displayed excellent convergent validity. We also hypothesized that the FAS-SR scores would be moderately associated with symptom severity, poor family function and functional impairment in OCD patients. Second, exploratory factor analysis was performed to explore the factor structure of the FAS-SR. We hypothesized that FA has multiple constructs rather than a single construct. Third, the study aimed to explore a mediation model in which FA mediated the association between symptom severity and functional impairment. We hypothesized that FA would mediate the relationship between OCD symptom severity and functional impairment.

## Materials and methods

### Participants

The translation and adaptation procedures of the family accommodation scale have been reported in our previous study (29). Additionally, the recruitment strategy for patients and corresponding relatives and the inclusion and exclusion criteria have been reported in detail (28). One relative was paired with each OCD patient in this study. Because information on 26 family members was lost, a total of 171 patients and 145 paired relatives were recruited from a specialized OCD outpatient clinic in Xiamen Xianyue Hospital from 2018 to 2020 for the present study. All patients and relatives provided informed consent before the beginning of the investigation, and the protocol of the study was reviewed by the Xiamen Xianyue Hospital ethics commitment (2018-KY-010).

### Measures

To ensure the stability of the result, the FAS-SR was first self-reported for the family member, and then, the trained interviewer evaluated the FAS-IR based on the blinded results of the FAS-SR. The instruments were detailed as follows. The assessments of patients and relatives were conducted in different rooms so that the relatives and OCD patients could respond without interference. The aim of the decision was to create a comfortable environment in which the relatives of OCD patients could thoroughly express and report their experiences of frustration or other negative emotions toward OCD patients. If a patient went to the clinic alone, the corresponding relative agreed to an interview at the next clinic in 7- to 10- days. The retest assessment of the FAS-SR was measured between 7 and 10 days in a partial sample.

A specifically created questionnaire was used to collect demographic and clinical variables for OCD patients, such as age, gender, educational level, marital status, occupational status, region, religion, age at the onset of symptoms, course period of illness and treatment, and family history of OCD. Demographic variables for family members included age, gender, educational level, marital status, and relationship with patients.

### Self-report measure

#### Family accommodation scale for obsessive compulsive disorder self-rated (FAS-SR)

The original version of the FAS-SR was developed by Pinto et al. to measure the frequency of FA in the past week based on the first section of the OCD symptoms checklist, which was self-reported by the patients’ relatives (25). The structure of the FAS-SR was identical to that of the FAS-IR, which included two sections, a symptom checklist and 19 items on accommodating behaviors. To help relatives more thoroughly understand and accurately comprehend their FA behaviors, some wording and the structuring of these FA items were modified in the FAS-SR (25). Some items from the FAS-IR that were originally evaluated by one item were individually divided into two items in the FAS-SR. For example, the item providing reassurance in FAS-IR was divided into two items about providing reassurance of obsession and compulsion. The FAS-SR item description and content were made clearer and more comprehensive, and more information and examples were provided in comparison to the FAS-IR. Consistent with the FAS-IR scoring method, the 19 items are scored on a 5-point Likert scale, and the responses are none, 1/week, 2–3/week, 4–6/week, and every day. The total score of the scale ranges from 0 to 76, and higher scores demonstrate more severe FA behaviors. The FAS-SR has been widely used in clinical and research settings, and has been adapted and translated into different languages (27, 30, 31). The average time of assessment of the FAS-SR was 24.42 ± 7.09 minutes.

#### Sheehan disability scale (SDS)

The SDS was administered to assess the patients’ functional impairment for all psychiatric disorders and is widely used in clinical and research settings (32). The SDS includes three domains: work/academic, social life/leisure, and family/home responsibilities. The total scores of the scales range from 0 to 30, and are measured on a visual analog scale as 0 (no impairment), 1–3 (moderated), 4–6 (moderated), 7–9 (marked), or 10 (extreme). The SDS has demonstrated good reliability and validity (32, 33).

#### Obsessive compulsive inventory revised (OCI-R)

The OCI-R is an 18-item scale used to evaluate OCD symptom dimensions in the past month for OCD patients (34–36). The scale includes six dimensions: obsessing, washing, checking, hoarding, neutralizing, and ordering symptoms. The total scores ranged from 0 to 72, and every item was measured on a 5-point scale from 0 to 4 (not at all, a little, moderately, a lot, and extremely). The scale displayed strong psychometric properties in OCD patients and non-clinical individuals (36). The OCI-R has also been widely used in OCD symptom assessment and improvement in both clinical practice and research settings (34, 37).

#### Zung self-rating depression scale (Zung SDS)

The scale is a 20-item self-report by patients about their depression (38). Every item of the scale is scored 1–4 (1 = a little of the time, 2 = some of the time, 3 = a good part of the time, 4 = most of the time). The Zung SDS is widely used in the clinic to evaluate some moods and conditions related to some patients with psychiatric disorders (38). The scale has displayed satisfactory psychometric features (39).

#### Family assessment device general functioning (FAD-12)

The FAD-12 was extracted from the original FAD and evaluates family functioning with 12 items for both patients and their relatives (40–42). The scale includes 6 forward-scored and 6 reverse-scored items, which measure responses on a scale from 1 to 4 for a total score of 12–48. Higher scores on the scale indicated worse levels of family functioning. The FAD-12 has been identified as a brief scale to measure family functioning with excellent reliability and validity (41).

### Clinical interview measure

#### Family accommodation scale interviewer-rated (FAS-IR)

The FAS-IR is a 12-item clinician-rated semistructured instrument that is regarded as the gold standard in measuring accommodating behaviors (18). The FAS-IR was first developed by Calvocoressi et al. and was revised and improved from the 13-item FAS in 1999 (18). The instrument includes two sections, the OCD symptom checklist and 12 items on accommodating behaviors. The first section includes eight kinds of obsession, seven kinds of compulsion, and five kinds of other OCD-related problems. The interviewer obtains information from the family member regarding the patient’s symptoms in the previous week and assesses the extent to which the family member participates in accommodating the patient’s symptoms. The second section elaborates on an OCD relative’s reports of the type of FA behaviors and the level of interference they engage in (18, 26). Each item includes common examples of accommodating behaviors, but the interviewers may wish to develop additional examples based on information collected from the relative’s report of the patient’s symptoms. The total scores of the scale range from 0 to 48, and responses are scored on a 5-point Likert scale (0 = none, 1 = 1/week, 2 = 2–3/week, 3 = 4–6/week, 4 = everyday; 0 = not at all, 1 = mild, 2 = moderate, 3 = severe, 4 = extreme). The FAS-IR has excellent psychometric properties and has been commonly used to evaluate the reduction in FA as a treatment target in studies of OCD patients’ family-based psychotherapy (13, 18, 26). The Chinese version of the FAS-IR was reported in 2021 and has satisfactory reliability and validity (28).

#### Clinical global impression of severity scale (CGI-S)

The CGI-S was extracted from the CGI and has a single item to assess the overall clinical severity of the patients’ symptoms and functional impairment (43). The total score ranges from 0 (healthy) to 6 (extremely or severe mental illness). The CGI-S was widely exploited in clinical and research settings, and the instrument had satisfactory properties in previous studies (43, 44).

#### Global assessment of functioning (GAF)

The GAF is a single item that measures the overall psychosocial and occupational functioning of individuals with a mental illness (45). The total score ranges from 1 to 100 and is divided into 10 equal intervals. A lower scale score shows worse global psychosocial function. The GAF is frequently used in both research and clinical settings and has adequate reliability and validity (45).

#### Yale-Brown obsessive compulsive scale (Y-BOCS)

The Y-BOCS is a 10-item instrument that evaluates OCD symptom severity in the past month and is regarded as the gold standard instrument to measure changes and improvement in severity during OCD treatment (46–48). The scale includes two subscales, with five items about obsessions and five items about compulsions. The scale is widely used in both clinical and non-clinical settings. The total scores range from 0 to 40, and every item is scored 0–4 (none, mild, moderate, severe, and extreme). The Y-BOCS has demonstrated satisfactory reliability and validity (46, 49). In the present study, the severity of OCD was assessed by the Y-BOCS based on the patient’s experience and the compulsive subscale based on the relative’s report.

### Statistical analyses

The level of agreement between family members’ observations and understanding of the OCD patients’ symptom dimensions on the FAS-IR and the FAS-SR was examined by the kappa coefficients. The item-level frequencies and Cronbach’s alpha coefficient were used to assess the reliability of the FAS-SR. The intraclass correlation coefficient (ICC) was calculated to evaluate the agreement between the FAS-SR and FAS-IR total scores. Exploratory factor analysis was employed to understand the factor structure of the FAS-SR. Primary components were extracted using varimax rotation, and eigenvalues were calculated to assess the amount of variance accounted for by a factor. The number of factors was determined based on both eigenvalues greater than 1 and screen plots. Two-way mixed consistency was used in the test-retest between the first and retested assessments of the FAS-SR. Spearman correlation coefficients were calculated to evaluate the convergent validity of the total FAS-SR scores associated with the FAS-IR, Y-BOCS, SDS, GAF, and FAD-12 scores based on the non-parametric distribution. The magnitude of associations between the total FAS-SR and FAS-IR scores on each of the criterion measures was compared by Steiger’s Z test (50).

Mediation analyses were performed to examine whether FA as measured on the FAS-SR mediated the relationship between symptom severity and functional impairment using the PROCESS macro for SPSS (51), which utilizes the bootstrapped standard errors method for the direct and indirect effects of the mediator variable. The basic information of this procedure is the same as the class Baron and Kenny method, but this approach was required to increase statistical power through bootstrapping procedures and take measures to specific tests for the mediated effect. The number of bootstrapped resamples was set at 5,000, and the indirect mediation effect was regarded as significant when the exclusion of zero was between the 95% confidence intervals.

All analyses were performed using the Statistical Package for the Social Science (SPSS) version 21.0. *P* < 0.05 was used to determine statistical significance.

## Results

### Frequency data for the family accommodation scale self-rated

A total of 171 patients and 145 paired relatives participated in the survey because 26 relatives did not complete the interview. Table 1 describes the demographic and clinical information of the participants. The age range of patients was 18–78 years old, with a mean age of 30.90 ± 10.61 years, and 54.4% were females. The relatives included 73 (50.3%) parents, 68 (46.9%) spouses and 4 (2.8%) others. The age range of relatives was 23–74 years old, with a mean age of 44.40 ± 10.54 years, and 53.8% were females. There were no significant differences in patient age, gender, or the total Y-BOCS scores of patients based on either patient or relative reports between relatives who completed the FAS-SR and those who did not (all *P* > 0.05).

**TABLE 1:** Demographic and clinical characteristics of participants.

Variables	Patients (*n* = 171)	Family members (*n* = 145)
Age (years) (Mean ± SD)	30.91 ± 10.61	44.40 ± 10.54
**Gender- n,%**		
Female	93 (54.4)	78 (53.8)
**Educational level- n,%**		
Primary school and below	8 (4.7)	15 (10.3)
Junior middle school	19 (11.1)	27 (18.6)
High school	53 (31.0)	39 (26.9)
College and above	91 (53.2)	64 (44.2)
**Marital status- n,%**		
Married	90 (52.6)	135 (93.1)
**Occupational status- n,%**		
Employed	68 (39.8)	93 (64.1)
Retired	3 (1.8)	15 (10.3)
Housewife	18 (10.5)	13 (9.0)
Unemployed	34 (19.9)	10 (6.9)
Student	41 (24.0)	1 (0.7)
Other	7 (4.1)	13 (9.0)
**Region- n,%**		
Urban	124 (72.5)	101 (69.7)
Suburban	8 (4.7)	7 (4.8)
Rural	39 (22.8)	37 (25.5)
Age at the onset of symptom (years) (Mean ± SD)	23.88 ± 10.73	–
Illness duration (years) (Mean ± SD)	7.04 ± 7.16	–
Treatment duration (years) (Mean ± SD)	2.47 ± 4.47	–
**Relationship with patient- n,%**		
Parents	–	73 (50.3)
Spouse	–	68 (46.9)
Other*	–	4 (2.8)

*Include adult child, sibling, and significant other.

Table 2 compares the agreement of relatives’ proportion of OCD symptom dimensions between the FAS-SR and FAS-IR. There was significant agreement on relatives’ proportion of types of OCD symptoms between the two scales, except for miscellaneous compulsions.

**TABLE 2:** The agreement in relatives’ endorsement of patient OCD symptom categories on FAS-IR vs. FAS-SR (*n* = 145).

Symptom dimension	FAS-IR		FAS-SR		Kappa	*P*
	n	100%	n	100%		
**Obsessions**						
Harming obsessions	62	42.8	58	40.0	0.574	<0.001
Contamination obsessions	89	61.4	83	57.2	0.686	<0.001
Sexual obsessions	2	1.4	2	1.4	–	–
Saving/losing obsessions	21	14.5	27	18.6	0.452	<0.001
Religious obsessions	14	9.7	15	10.3	0.349	<0.001
Obsession with need for symmetry or exactness	28	19.3	46	31.7	0.360	<0.001
Somatic obsessions	36	24.8	40	27.6	0.501	<0.001
Miscellaneous obsessions	51	35.2	71	50.0	0.445	<0.001
**Compulsions**						
Cleaning/washing compulsions	101	69.7	98	67.6	0.664	<0.001
Checking compulsions	82	56.6	78	53.8	0.554	<0.001
Repeating rituals	46	31.7	49	33.8	0.546	<0.001
Counting compulsions	12	8.3	15	10.3	0.225	0.006
Ordering/arranging compulsions	19	13.1	14	9.7	0.693	<0.001
Saving/collecting compulsions	7	4.8	8	5.5	0.508	<0.001
Miscellaneous compulsions	56	38.6	78	53.8	0.241	0.002

Table 3 displays the frequency data for items on the FAS-SR. In sum, the proportion of participants who endorsed at least one, and daily (or an extreme) type of accommodating behavior in the past week was 97.9 and 56.6%, respectively. Both the provision of reassurance associated with obsessions (71.7%) and the reduction of leisure time (67.6%) were the most common phenomena. In addition, approximately half of the relatives believed that they provided reassurance about compulsions (59.3%), avoided talking about OCD triggers (62.8%), stopped themselves from doing things that could trigger OCD behaviors (54.5%), did not stop unusual OCD-related behaviors (53.8%), and changed their work/school schedules (53.1%). The least frequently endorsed accommodating behaviors included helping patients prepare food (29.0%), making it possible for patients to perform compulsions (23.4%), and providing items needed to perform compulsions (22.6%).

**TABLE 3:** The percentage of FAS-SR items.

FAS-SR items	Mean ± SD	Item-total r	Alpha if removed	Range	Frequency of endorsement					Percentage^a^
					0	1	2	3	4	
1. Reassured patient that there were no grounds for OCD concern	1.77 ± 1.47	0.481	0.868	0–4	41 (28.3)	27 (18.6)	27 (18.6)	24 (16.6)	26 (17.9)	104 (71.7)
2. Reassured patient that compulsions took care of OCD concern	1.43 ± 1.49	0.466	0.868	0–4	59 (40.7)	24 (16.6)	26 (17.9)	13 (9.0)	23 (15.9)	86 (59.3)
3. Waited for patient	1.26 ± 1.52	0.362	0.869	0–4	75 (51.7)	15 (10.3)	18 (12.4)	17 (11.7)	20 (13.8)	70 (48.3)
4. Directly participated in compulsions	0.99 ± 1.49	0.480	0.867	0–4	91 (62.8)	12 (8.3)	15 (10.3)	6 (4.1)	21 (14.5)	54 (37.2)
5. Made it possible for patient to complete compulsions	0.48 ± 0.99	0.555	0.866	0–4	111 (76.6)	12 (8.3)	14 (9.7)	3 (2.1)	5 (3.4)	34 (23.4)
6. Provided items needed to perform compulsions	0.47 ± 1.01	0.460	0.869	0–4	112 (77.2)	13 (9.0)	10 (6.9)	5 (3.4)	5 (3.4)	33 (22.6)
7. Made it possible for patient to avoid OCD triggers	0.94 ± 1.39	0.390	0.869	0–4	88 (60.7)	18 (12.4)	15 (10.3)	8 (5.5)	16 (11.0)	57 (39.3)
8. Helped patient make simple decisions	0.68 ± 1.06	0.435	0.868	0–4	91 (62.8)	25 (17.2)	17 (11.7)	8 (5.5)	4 (2.8)	54 (37.2)
9. Helped patient with personal tasks	0.56 ± 1.14	0.430	0.869	0–4	110 (75.9)	11 (7.6)	10 (6.9)	6 (4.1)	8 (5.5)	35 (24.1)
10. Helped patient prepare food	0.77 ± 1.36	0.405	0.868	0–4	103 (71.0)	11 (7.6)	3 (3.3)	10 (6.9)	14 (9.7)	42 (29.0)
11. Took on patient’s family or household responsibilities	1.10 ± 1.50	0.374	0.869	0–4	85 (58.6)	10 (6.9)	8 (8.8)	10 (6.9)	20 (13.8)	60 (41.4)
12. Avoided talking about OCD triggers	1.68 ± 1.62	0.405	0.872	0–4	54 (37.2)	23 (15.9)	8 (8.8)	16 (11.0)	34 (23.4)	91 (62.8)
13. Stopped self from doing things that could trigger OCD	1.50 ± 1.63	0.429	0.868	0–4	66 (45.5)	18 (12.4)	8 (8.8)	15 (10.3)	31 (21.4)	79 (54.5)
14. Made excuses or lied for patient to cover up OCD	0.50 ± 0.92	0.273	0.874	0–4	100 (69.0)	28 (19.3)	5 (5.5)	3 (2.1)	4 (2.8)	45 (31.0)
15. Didn’t stop unusual OCD-related behaviors	1.37 ± 1.53	0.304	0.871	0–4	67 (46.2)	17 (11.7)	15 (16.5)	10 (6.9)	25 (17.2)	78 (53.8)
16. Put up with unusual conditions in home due to OCD	1.06 ± 1.49	0.324	0.871	0–4	88 (60.7)	8 (5.5)	11 (12.1)	8 (5.5)	20 (13.8)	57 (39.3)
17. Cut back on leisure time	1.51 ± 1.42	0.549	0.870	0–4	47 (32.4)	35 (24.1)	14 (15.4)	14 (9.7)	22 (15.2)	98 (67.6)
18. Changed my work/school schedule	1.12 ± 1.36	0.560	0.868	0–4	68 (46.9)	32 (22.1)	10 (11.0)	7 (4.8)	17 (11.7)	77 (53.1)
19. Put off my own family responsibilities	0.83 ± 1.22	0.442	0.869	0–4	84 (57.9)	30 (20.7)	10 (11.0)	8 (5.5)	10 (6.9)	98 (42.1)

FAS-SR, Family Accommodation Scale for Obsessive-compulsive Disorder, Self-reported. 0 = none/never, 1 = 1 day, 2 = 2–3 days, 3 = 4–6 days, 4 = every day. ^a^ Percent of respondents reporting frequency of accommodation as “often-at least once per day” or greater (≥1).

The total FAS-SR score ranged from 0 to 68, and the mean of the total scores was 20.01 ± 14.39.

### The factor structure of the family accommodation scale self-rated

There were five factors with eigenvalues greater than 1 (6.065, 1.716, 1.434, 1.209, and 1.067). According to the results of the screen plot and the eigenvalue figures, the three factors of the scale were more reasonable and were finally identified. Moreover, both Bartlett’s test was 940.427 (*df* = 171, *P* < 0.001), and Kaiser-Meyer-Olkin (KMO) was 0.844, indicating that the sample was appropriate for describing factor analysis. The cumulative contribution rate was 48.50%. The scale included three factors: (1) providing reassurance, participation, (2) facilitation, and (3) modification. The details are described in Table 4.

**TABLE 4:** Exploratory factor analysis of the Chinese version of the FAS-SR.

FAS-SR items	Factor 1	Factor 2	Factor 3
5. Made it possible for patient to complete compulsions	**0.753**	0.176	0.175
6. Provided items needed to perform compulsions	**0.693**	0.205	0.017
7. Made it possible for patient to avoid OCD triggers	**0.675**	0.150	0.019
4. Directly participated in compulsions	**0.644**	0.058	0.272
2. Reassured patient that compulsions took care of OCD concern	**0.618**	0.064	0.223
3. Waited for patient	**0.604**	0.204	0.047
1. Reassured patient that there were no grounds for OCD concern	**0.602**	0.072	0.242
15. Didn’t stop unusual OCD-related behaviors	**0.498**	0.203	0.124
8. Helped patient make simple decisions	**0.473**	0.393	0.155
9. Helped patient with personal tasks	0.286	**0.712**	-0.015
10. Helped patient prepare food	0.193	**0.701**	0.165
11. Took on patient’s family or household responsibilities	0.139	**0.637**	0.259
14. Made excuses or lied for patient to cover up OCD	-0.023	**0.628**	0.154
16. Put up with unusual conditions in home due to OCD	0.320	**0.484**	0.049
13. Stopped self from doing things that could trigger OCD	0.227	**0.464**	0.356
17. Cut back on leisure time	0.118	0.061	**0.859**
18. Changed my work/school schedule	0.179	0.172	**0.789**
19. Put off my own family responsibilities	0.281	0.149	**0.611**
12. Avoided talking about OCD triggers	0.090	0.284	**0.570**
Cronbach’s	0.826	0.741	0.746

FAS-SR, Family Accommodation Scale for Obsessive-compulsive Disorder, Self-rated.

The factor loadings ≥ 0.40 are marked in the bold.

### Reliability and validity

The FAS-SR demonstrated Cronbach’s alpha of 0.879, and the corresponding Cronbach’s alpha of three factors were 0.826 (factor 1), 0.741 (factor 2) and 0.746 (factor 3), respectively. Additionally, the total FAS-IR score ranged from 0 to 44, with a mean of 13.49 ± 8.24. The ICC between the FAS-SR and FAS-IR scores was 0.795 (95% CI, 0.715–0.852).

A total of 16 relatives were evaluated to measure the test-retest reliability. The ICC was 0.97 (95% CI: 0.92–0.99) between the first assessment (P50:28, P25-P75:7–38) and retest assessment (P50:25, P25-P75:6.25–30). There were no statistically significant differences in age, gender, or Y-BOCS total scores of OCD patients rated by relatives’ reports between the relatives who completed and did not complete the retest of the FAS-SR (all *P* > 0.05).

A higher level of FA was significantly associated with more severe symptom severity in OCD patients measured by the Y-BOCS based on relative reports (*r*_*s*_ = 0.327, *P* < 0.05) but was slightly significantly associated with patient reports (*r*_*s*_ = 0.188, *P* = 0.023). In addition, a higher total FAS-SR score was associated with a worse level of family function (*r*_*s*_ = 0.157, *P* = 0.060 for patient interview, *r*_*s*_ = 0.342, *P* < 0.001 for relative-rated), a higher level of functional impairment (*r*_*s*_ = 0.286, *P* < 0.001), OCI-washing (*r*_*s*_ = 0.357, *P* < 0.001), OCI-ordering (0.181, *P* = 0.030), and a lower GAF score (*r*_*s*_ = -0.399, *P* < 0.001). There was no statistical association between the FAS-SR scores and Zung SDS scores (*r*_*s*_ = 0.048, *P* = 0.563). The results of Steiger’s Z test demonstrated that there was no significant difference between the FAS-IR and FAS-SR on each of the criterion instruments. The results are displayed in Table 5.

**TABLE 5:** Convergent validity of FAS-SR with criterion measures as compared to FAS-IR.

	FAS-SR total	*P*	FAS-IR total	*P*	Steiger’s Z^a^
**Patients rated (*n* = 171)**					
Y-BOCS total	0.188	0.023	0.289	<0.001	−1.756
Patient obsession severity	0.140	0.092	0.214	<0.001	−1.267
Patient compulsion severity	0.207	0.013	0.298	<0.001	−1.589
Patient global functioning (GAF)	−0.399	<0.001	−0.433	<0.001	0.637
Functioning impairment (SDS)	0.286	<0.001	0.300	<0.001	−0.248
Work/school	0.133	0.112	0.160	0.054	−0.460
Social life	0.290	<0.001	0.285	0.001	0.088
Family life/home responsibility	0.315	<0.001	0.344	<0.001	−0.521
Family global functioning (FAD)	0.157	0.060	0.158	0.057	−0.017
OCI total score	0.155	0.062	0.157	0.060	−0.034
OCI hoarding	0.107	0.217	0.013	0.878	1.583
OCI ordering	0.181	0.030	0.177	0.033	0.069
OCI checking	0.060	0.470	−0.036	0.666	0.404
OCI neutralizing	−0.025	0.767	0.013	0.876	−0.639
OCI obsessing	−0.036	0.669	−0.036	0.664	0.000
OCI washing	0.357	<0.001	0.414	0.000	−1.051
Zung SDS	0.048	0.563	0.134	0.108	−1.464
**Relative rated (*n* = 145)**					
Patient compulsion severity (Y-BOCS)	0.332	<0.001	0.436	<0.001	−1.944
FAS-IR total	0.749	<0.001	–	–	–
Family global functioning (FAD)	0.342	<0.001	0.373	<0.001	−0.564

^a^Two-tailed Z-critical is 1.96 for *P* < 0.05 and 2.58 for *P* < 0.01. FAS-SR, Family Accommodation Scale for Obsessive-compulsive Disorder, Self-rated. FAS-IR, Family Accommodation Scale for Obsessive-compulsive Disorder, Interviewer-Rated.

### Mediation of the relationship between symptom severity and functional impairment by family accommodation

**FIGURE 1:**
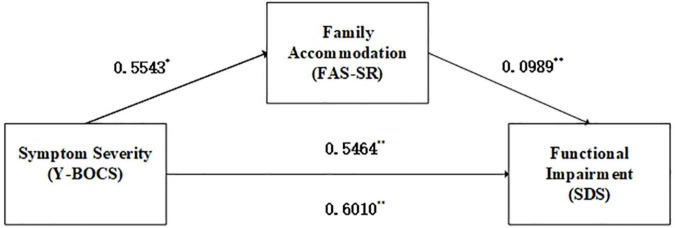
Mediation effects of family accommodation on symptom severity and functional impairment. *P < 0.05, **P < 0.01. Y-BOCS, Yale-Brown Obsessive-Compulsive Scale; FAS-SR, Family Accommodation Scale for OCD Self-rated version; SDS, Sheehan Disability Scale.

This model examined whether FA was a mediator variable to measure the relationship between symptom severity on the clinically administered Y-BOCS and functional impairment, controlling for patient age, gender, educational level, marital status, occupational status and region. The results demonstrated that FA significantly and independently mediated the association between symptom severity and functional impairment (a*b path, β = 0.0548, 95% CI: 0.0033–0.1270). Higher symptom severity was associated with higher FA score, and FA score was positively associated with functional impairment. The direct effect of symptom severity on OCD functional impairment remained significant after the inclusion of mediators (c’ path, β = 0.5464, *SE* = 0.1101, *P* < 0.001). Figure 1 illustrates the meditation model.

## Discussion

To the best of our knowledge, this is the first study to report FAS-SR in adult OCD patients in China. Similar to previous reports, this study demonstrated that the Chinese version of the FAS-SR has satisfactory reliability and validity. The Chinese version of the FAS-SR can be widely used in Chinese OCD participants to assess and quantify family members’ responses to the symptoms of their loved ones.

Consistent with previous research, family members reported high rates of FA, again confirming that FA is believed to be a common and ubiquitous phenomenon in Chinese family members of OCD patients (7–9, 18, 27, 30, 52). The results demonstrated that almost all subjects endorsed at least one kind of FA behavior, and more than half of the participants endorsed every day or had facilitated an extreme FA behavior within the past week. These results are also consistent with the original version in a previously reported study (25). Although the behaviors may be seemingly relatively innocuous, they unfortunately caused undesired consequences of symptom maintenance and reinforced OCD symptomology in the long run. The family members aimed to help the patients feel safe by relieving their in-the-moment anxiety and distress and not to disrupt daily life routines or time spent executing compulsions. However, FA was usually detrimental to the patients’ long-term mental health and function by preventing OCD patients from habituating to anxiety and learning that the consequences they feared typically did not occur. As a result, the finding of a high incidence of FA affirmed that it is necessary to focus on the important role of FA in OCD occurrence, development, and outcome.

The most frequent FA behavior was offering reassurance about continued obsessions and cutting back on leisure time. Consistent with previous studies, the provision of reassurance related to obsessions also confirmed that this item was the most common type of accommodation (7, 8, 25, 27, 30, 52, 53). Compared to other obvious behaviors, this method of accommodation was perceived as more passive and with less direct involvement and participation, so this behavior was more common in relatives of OCD patients. On the other hand, making it possible for patients to perform compulsions and providing items needed to do compulsions were less frequently reported. There are more overt tasks that family members need to direct to take part in some compulsions. Overt tasks benefit from increased focus on direct family involvement compared to providing assurance, needing more time and increasing the burden.

Considering the high incidence of FA and common behaviors in relatives of OCD patients, it may be that professional policies should be developed to target these myriad behaviors and integrate the relatives into the treatment plan on evidence-based relative management strategies to help the patients with OCD better tackle OCD-related distress and anxiety with self-efficacy. Additionally, providing and popularizing some knowledge on proper psychoeducation about the deleterious consequences of FA, integrating family members into the patients’ treatment and training them on appropriate responses to OC symptoms would increase family support and eliminate maladaptive behaviors.

As expected, the result of the test-retest analysis was excellent. To the best of our knowledge, this is the first study to explore the test-retest reliability of the FAS-SR. These results reinforced the stability of the self-reported instrument for assessing FA. In addition, internal consistency was similar to the original and other language versions of the FAS-SR, which reported coefficients of 0.90, 0.88, and 0.936, respectively, demonstrating strong internal consistency of the instrument (25, 27, 30). The results confirmed that the Chinese version of the FAS-SR had satisfactory reliability.

Because the sample size of the study taking the original version of the FAS-SR was too small, the factor structure of the FAS-SR was not explored (25). This hinders the contradistinction compared to the original version of the FAS-SR. Our result was not consistent with the Hindi versions of the FAS-SR (30). The reasons for the difference between the Chinese and Indian language versions of the FAS-SR are as follows. Owing to some cultural differences between the two countries and the differences in the inclusion criteria for participants in the two studies, these discrepancies may explain the different factor structures of the Chinese and Hindi versions of the FAS-SR. Additionally, the multiple structure of the FAS-SR showed that the assessment of FA required the consideration and analysis of these problematic behaviors from different dimensions and aspects. In conclusion, the Chinese version of the FAS-SR displayed multiple structures, not a global structure.

The hypothesis that the total FAS-SR score was moderately correlated with several variables related to patient symptom severity and OCD-related family pathology was supported. In addition, the association between the FAS-SR score and observed variables did not differ from the association between the FAS-IR scores and the same observed variables (27, 52). The results were consistent with those reported by Pinto et al. in the original version of the FAS-SR (25). Moreover, our results are in accordance with previous reports that demonstrated severe FA behaviors related to poorer family functioning, higher symptom severity, and more severe functional impairment (52, 53). These results suggested that dysfunctional family interactions, family conflict and distress due to the home environment described FA behaviors.

Consistent with the hypothesis, the total score of the FAS-SR was significantly associated with OCD symptom severity in both patient-rated and relative reports, and the relationship was very weak. This result was consistent with the majority of previous reports, especially from a recent meta-analysis (25, 30), and it was likely that OCD patients who displayed higher symptom severity demand increased FA behavior. However, the result was inconsistent with a study of the Japanese population reported in 2016 (27). Compared to the association between the total FAS-IR scores and patient-rated OCD symptom severity, the figure was relatively lower than the abovementioned results, even though the difference was not statistically significant. There was a possibility that the relatives of OCD patients underestimated their accommodating behaviors by self-reporting despite the existence of severe OCD symptoms. Moreover, family members may believe their accommodating behavior is simply supportive of OCD patients. Additionally, the reported high levels of shame, embarrassment and stigma attached to OCD often result in the patients intentionally ignoring and decreasing OCD symptom severity. In addition, it should be emphasized that the relationship between OCD symptom severity and FA is likely bidirectional, necessitating future longitudinal investigations to understand its clinical course.

Similar to previous studies, in regard to the clinical correlates of FA in OCD, poor family functioning, washing symptoms, higher CGI-S scores, and lower GAF scores were significantly correlated with the total FA score (25, 27, 30, 50). These results supported the hypothesis that some factors were significantly associated with the total FA scores, and the FAS-SR had good convergent validity. The symptom of OCI washing was the most common symptom reported. This result may have application in the clinic, especially when doctors encounter patients who have this primary symptom. However, some other symptoms were not associated with FA, which is particularly true if the patient struggles with sharing behaviors perceived as grotesque or amoral, making him or her less prone to seek accommodation from family members. Owing to the limitation of the research design, it was not obvious that the family members had such psychopathologies before or after the onset of OCD symptoms. There was no statistical association between the FAS-SR and Zung SDS scores, and the results showed that the FAS-SR displayed excellent discrimination validity.

Inconsistent with the hypothesis, FA partially mediated the relationship between symptom severity and functional impairment. Similar to previous reports, the mediation model demonstrated that more severe OCD symptomology was linked with increased FA behaviors, which ultimately caused greater functional impairment in OCD patients (9). Contrary to the hypothesis, the direct effect of symptom severity on functional disability-associated OCD remained significant in the mediation model. Piacentini et al. also reported a change in FA before the change in OCD symptom severity and functional impairment, suggesting the importance of reducing accommodating behaviors to decrease symptom severity and functional impairment (54). Therefore, regarding their respective contributions to functional impairment in OCD patients, the results showed the importance of identifying FA behaviors and targeting symptom severity. In conclusion, it is important to target these FA behaviors in OCD evaluation and treatment. As such, family-based treatments designed to target these specific symptoms and integrate family members in the therapeutic process are expected to be particularly efficacious.

Based on the study design and other factors, future research should explore the shortcomings related to this study. First, because the FAS-SR and FAS-IR had differences in the number of items and the response options, the FAS-SR was developed based on the structure of the FAS-IR. As a result, there was no way to compare each individual item between the FAS-SR and FAS-IR. Second, our study design was not designed to evaluate the sensitivity of FA to changes in treatment. It is necessary to conduct follow-up studies to understand the relationship between the accommodating behavior and treatment outcome of OCD in future studies. Third, these findings should be considered within the limitations of developing a meditation model in a cross-sectional design. Fourth, the FAS-SR reported that one’s own behavior was susceptible to certain biases and different levels of understanding, and it was limited to people with low levels of education. Because the sample of other relatives was small, the type of kinship should be diversified and balanced to assess how this variable affects the extent of accommodating behaviors in future studies. In addition, the sample of the present study was insufficient to explore the factor structure of the FAS-SR with confirmatory factor analysis; future studies should include larger sample sizes to further understand the factor structure of the FAS-SR.

## Conclusion

In sum, the FAS-SR could overcome the limitations of interviewer administration and systematically evaluate problematic behaviors based on the relatives’ view and understanding. The FAS-SR provided the opportunity to target FA behaviors through relative self-report, which may be beneficial for reducing clinicians’ time, saving labor costs and speeding up the diagnostic process compared to the use of clinician-administered instruments. Additionally, the present study filled a current gap in the literature by establishing a self-reported instrument for relatives of OCD patients that enables a standardized method of assessing FA behaviors in OCD patients and has some implications for clinical assessment, intervention and academic areas in China. First, the FAS-SR may be a cost- and time-effective instrument to evaluate the involvement of family members in OCD patients’ symptoms, which could help clinicians identify the level of accommodation and obtain more detailed information on family behaviors. Second, given the high incidence of FA behaviors reported in this study and linking FA with family functioning, symptom severity, and functioning impairment, it seems that the evaluation of FA behaviors should be incorporated into all pretreatment assessments of OCD to help guide clinicians in the formulation of family-based treatment plans. Third, having more detailed information about the most common type of accommodating behaviors guides clinicians in their assessment of family dynamics, providing more specific psychoeducation and enabling the development of exposures and other desirable strategies to reduce FA behaviors. Fourth, FA partially mediated the relationship between symptom severity and functional severity. Given the association with decreased function and poorer treatment response, targeted intervention and treatment for those associations and construal are expected to improve the treatment outcome of OCD patients. In sum, these results demonstrated that the Chinese version of the FAS-SR has sound psychometric properties, which suggests that the instrument is a useful tool to measure FA and could aid in early treatment intervention and personalized treatment efforts in the future.

## Data availability statement

The raw data supporting the conclusions of this article will be made available by the authors, without undue reservation.

## Ethics statement

The studies involving human participants were reviewed and approved by the Xiamen Xianyue Hospital Ethics Commitment. The patients/participants provided their written informed consent to participate in this study.

## Author contributions

ZL, WZ, and LD designed the protocol in this study. ZL, CY, YC, and LD collected the data and performed the clinical assessment. ZL, CY, and YC analyzed the data. ZL wrote the manuscript. All authors contributed to and have approved the final manuscript.
